# Association Between HLA Class II Gene Polymorphisms and Cytokine Levels in PLWH with HIV-Related Dermatoses in Latvia

**DOI:** 10.3390/medicina61061091

**Published:** 2025-06-15

**Authors:** Alena Soha, Inga Azina, Maksims Zolovs, Darja Arina Miskina, Viktorija Murasko, Baiba Rozentale, Ilona Hartmane, Andris Rubins

**Affiliations:** 1 Doctoral Program, Riga Stradiņš University, LV-1007 Riga, Latvia; 2 Latvian Center for Infectious Diseases, Riga East University Hospital, LV-1038 Riga, Latvia; inga.azina@aslimnica.lv (I.A.); baiba.rozentale@rsu.lv (B.R.); 3 Statistics Unit, Riga Stradiņš University, LV-1007 Riga, Latvia; maksims.zolovs@rsu.lv; 4 Institute of Life Sciences and Technology, Daugavpils University, LV-5401 Daugavpils, Latvia; 5 Residency Program, University of Latvia, LV-1586 Riga, Latvia; darja.miskina@inbox.lv (D.A.M.); victoria.murasko@gmail.com (V.M.); 6 Department of Dermatology and Venerology, Riga Stradiņš University, LV-1007 Riga, Latvia; ilona.hartmane@1slimnica.lv; 7 Faculty of Medicine, University of Latvia, LV-1586 Riga, Latvia; arubins@apollo.lv

**Keywords:** HIV, skin disorders, DQA1, DQB1, DRB1, cytokines

## Abstract

*Background and Objectives:* This study explores the immunogenetic associations of human leukocyte antigens (HLAs) and cytokine levels in people living with HIV/AIDS (PLWH) who exhibit HIV-related skin disorders. HIV-related skin disorders, including inflammatory eruptions, atopic and seborrheic dermatitis, psoriasis, and pruritic papular eruption, are common among PLWH. These conditions may be influenced by genetic and immunological factors. This study aims to investigate the associations between HLA class II alleles, cytokine levels, and the presence of HIV-related dermatoses, providing insights into genetic susceptibility and immune mechanisms. *Materials and Methods:* This study included 115 PLWH with HIV-related skin disorders and a control group of 80 healthy individuals. HLA allele frequencies were analyzed, and cytokine levels (IL-1β, IL-10, IFN-y) were measured in blood samples. Statistical analyses were performed to identify significant differences in allele frequencies and cytokine responses between the groups. *Results:* Risk alleles (DQB1*0201:0301, OR = 19.4 and DQA1*0101:0501, OR = 4.2) and protective alleles (DRB1*07:13, OR = 0.19, DRB1*01:13, OR = 0.09, DRB1*04:11, OR = 0.07, and DQA1*0501:0501, OR = 0.24) showed statistically significant differences in frequency (*p* < 0.05) between PLWH and healthy controls. The protective DQA1*0501:0501 allele was associated with elevated levels of IL-1β (*p* < 0.001, r = 0.79) and IL-10 (*p* = 0.010, r = 0.63). Increased IL-1β levels may enhance immune responses, while higher IL-10 levels may exert anti-inflammatory effects, potentially reducing susceptibility to HIV-related dermatoses. Regression analysis revealed that IL-1β (Exp(B) = 0.76, *p* < 0.001) and IFN-γ (Exp(B) = 1.06, *p* = 0.043) are significant predictors for the likelihood of developing HIV-related dermatoses. An increase in IL-1β levels reduced this likelihood by 24%, while an increase in IFN-γ levels increased it by 6%. *Conclusions:* The findings emphasize the interaction between HLA class II alleles and cytokine profiles in determining genetic risk and clinical outcomes in PLWH with HIV-related skin disorders. Protective alleles, such as DQA1*0501:0501, may contribute to immune regulation, offering potential targets for therapeutic interventions in PLWH with dermatoses. Our results highlight the importance of IL-1β and IFN-γ as key modulators in the progression of HIV infection and the development of HIV-related dermatoses. Further research is needed to explore the mechanisms underlying these associations, particularly in the Latvian population, to inform targeted therapeutic strategies.

## 1. Introduction

The ongoing HIV epidemic, combined with the availability of more effective treatments, has led to an increasing number of PLWH. Skin manifestations associated with HIV/AIDS or its therapies occur in up to 95% of patients and are among the most frequent manifestations of the infection [1,2].

### 1.1. Cytokines

Cytokine profiling enables the evaluation of immune regulation defects in PLWH with dermatological manifestations. Cytokines—polypeptides with diverse biological functions—play key roles in physiological and pathological processes, including inflammation, autoimmune diseases, and angiogenesis [3].

### 1.2. IL-10

IL-10 protects the host from systemic inflammation following toxin-induced injury but, paradoxically, has been shown to increase susceptibility to lethal infections in various experimental models [4]. Elevated IL-10 levels are observed in late-stage PLWH, contributing to immunosuppression and a higher risk of secondary infections [5,6]. IL-10 deregulation has been implicated in the development of psoriasis, systemic lupus erythematosus, and allergies [7]. IL-10 plays a critical role in mitigating the excessive immunopathology and tissue damage caused by the overproduction of inflammatory cytokines and is central to regulating tissue remodeling during wound healing [8].

### 1.3. IFN-γ

IFN-γ plays a critical role in antimicrobial and antitumor immunity. It enhances antigen (Ag) presentation through MHC class I and class II molecules, regulates various genes, and facilitates proapoptotic responses in infected cells [9,10].

IFN-γ levels have been reported to be elevated in PLWH on HAART (highly active antiretroviral therapy). Studies have shown that IL-6, IFN-γ, and TNF-α are elevated in PLWH. IL-6 and TNF-α levels decrease with consistent HAART use, whereas serum IFN-γ levels tend to increase with continued HAART therapy [10].

IL-17, IFN-γ, IL-22, and TNF are key mediators in the regulation of keratinocyte activity, promoting proliferation and stimulating the production of chemokines, cytokines, and antimicrobial peptides (AMPs). These molecules establish a feedback loop, wherein keratinocyte-derived products interact with dendritic cells (DCs), T cells, and neutrophils, thereby sustaining the inflammatory process in the skin [11]. IFN-γ plays a dual role in immune regulation, contributing to immune homeostasis while also being implicated in the progression of autoimmune and inflammatory disorders. Its dysregulation can exacerbate clinical symptoms and pathological changes in conditions such as psoriasis and atopic dermatitis [12].

In psoriasis, skin-resident T cells can produce IFN-γ, IL-17, or IL-22 in response to specific activation stimuli or antigens. These cytokines drive the production of chemokines that amplify immune responses, with Th1 activation particularly enhancing IFN-γ production [11].

Recent studies have demonstrated the therapeutic potential of IFN-γ-induced mesenchymal stem cell extracellular vesicles (IFN-γ-iMSC-EVs) in atopic dermatitis (AD). These vesicles suppress Th2 cytokine receptor expression and downstream signaling while also restoring epidermal barrier integrity and lipid synthesis, offering a promising approach to mitigating AD-related skin damage [9].

### 1.4. IL-1β

Although IL-1β is primarily considered an inflammatory mediator, several findings have highlighted its role in maintaining the homeostasis of cells, tissues, and organs [13,14].

Recent studies have indicated that IL-1 family molecules possess both homeostatic and inflammation-related defensive functions [15], and IL-1β is present in the bloodstream in both healthy and disease conditions [16].

Patients with seborrheic dermatitis (SD) have demonstrated elevated levels of IL-1α, IL-1β, TNF-α, IFN-γ, IL-12, and IL-14 [17]. Individuals with psoriasis exhibit increased levels of IL-1, IL-17A, and IL-36, compared to those with healthy skin [18].

### 1.5. HLA and Cytokines

Previous studies have reported that specific HLA class I alleles are associated with the rate of HIV progression [19]. Variations in HLA alleles have been linked to differing outcomes of HIV [20]. HLA molecules play a crucial role in shaping cytokine profiles, which may predispose individuals to autoimmune diseases. The DRB1*0401 and DQ8 alleles activate CD4+ T cells, resulting in the production of Th1/Th17 cytokines, as well as IL-13 and IL-10 [21]. The class I HLA alleles A*0201 and A*6801 have also been associated with the secretion of IFN-γ and IL-10 [22].

One study linked the HLA DRB1*03 allele to the Epstein–Barr virus (EBV) viral load, as well as IL-4 and IL-6 levels, in PLWH [23]. Another associated the HLA-B14-C08 haplotype with elevated IL-10 levels in cervical disease, suggesting a connection between HLA class I alleles and cytokines in cervical carcinogenesis [24].

No studies have yet examined the relationship between HLA gene variability and serum cytokine expression in PLWH with dermatological disorders. This study aims to explore the potential correlation between HLA gene polymorphisms and cytokine expression.

## 2. Materials and Methods

This study aimed to identify genetic markers associated with immunological status by evaluating the serum levels of IL-1β, IFN-γ, and IL-10; CD4+ cell counts; and HIV RNA viral load detection at the onset of dermatological disorders, as well as comparing genetic polymorphisms and cytokine levels between a group of PLWH with skin disorders and a healthy control group.

### 2.1. Study Design

A combined retrospective and prospective study was conducted between 2022 and 2023 at Riga East University Hospital, the Latvian Center for Infectious Diseases, and the Joint Laboratory of Clinical Immunology and Immunogenetics at Riga Stradiņš University (RSU/JLCII).

The study protocol received ethical approval from the Latvian Central Medical Ethics Committee (approval code: 01-29.1.2/1670) and the Genetic Council (approval code: A-3/22-05-09) for genetic analysis. Prior to participation, written informed consent was obtained from all individuals or their legal representatives, ensuring compliance with ethical standards for clinical research.

This investigation builds upon earlier research into HIV-related skin disorders among PLWH. These conditions include pruritic papular eruption (PPE), xerosis with secondary infections, atopic dermatitis (AD), seborrheic dermatitis (SD), prurigo nodulari, eosinophilic folliculitis, psoriasis, and adverse drug reactions linked to antiretroviral therapy (ART).

PLWH with infectious or neoplastic opportunistic conditions, as well as those with active TB, were excluded from the analysis.

### 2.2. Study Population

The research group consisted of 115 PLWH with HIV-related skin disorders (mean age, 48 years; 31% women and 69% men) in Latvia. A total of 70 patients in the cohort group had a previous history of TB, reflecting the high prevalence of co-infections in HIV-positive populations. This factor was considered during the analysis, as it could potentially impact the observed associations between HLA alleles and cytokine production.

All patients with HIV-1 infection were prescribed antiretroviral therapy (ART) in accordance with the European Guidelines for the treatment of HIV-infected adults; however, some patients exhibited insufficient adherence to ART due to their socioeconomic status.

The control group comprised 80 healthy individuals (mean age, 52 years; 40% women and 60% men), all of whom were free from HIV-1 infection, tuberculosis (TB), and any skin disorders.

### 2.3. Genetic Analysis

As described in our previous study [25], HLA class II genotype analysis for each patient was conducted at the RSY/JLCII.

Peripheral blood samples (5 mL) were collected in tubes containing EDTA and stored at −20 °C until further processing. DNA extraction was performed using the QIAamp^®^ DNA Blood Kit (QIAGEN, Hilden, Germany), following the standard protocol provided by the manufacturer. For DNA amplification, polymerase chain reaction (PCR) was employed using low-resolution sequence-specific primers (DNA-Technology, Moscow, Russia), adhering to the manufacturer’s guidelines for optimal performance.

This method is widely recognized for its cost efficiency and reliability in both clinical and research applications, particularly for low- to intermediate-resolution typing. Given that the study emphasizes broader HLA associations rather than detailed polymorphic variations, PCR-SSP was selected as a suitable approach to effectively address the key research questions. Internal and positive controls were included in each kit to ensure the quality of the PCR process. HLA typing targeted the identification of alleles for HLA-DRB1* (01 to 18), HLA-DQA1* (01:01, 01:02, 01:03, 01:04, 02:01, 03:01, 04:01, 05:01, 06:01), and HLA-DQB1* (02:01–02:02, 03:01–04, 04:01–04:02, 05:01–04, 05:02–03, 06:01, 06:02–08).

PCR amplification was carried out using the DT-Lite Real-Time PCR Thermal Cycler (DNA-Technology, Moscow, Russia), with the reaction mixture undergoing 35 cycles.

A final extension step was included at the end of the 35 cycles to ensure complete amplification. The primers used in the reaction had a melting temperature of approximately 65–67 °C, optimized for the annealing step to enhance specificity and efficiency.

To reduce variability related to ethnic and geographic factors, participants for both the research and control groups were selected from the same region, ensuring no familial connections between them. The control group consisted of healthy individuals whose families had resided in the region for at least three generations. This approach ensured that the allele distribution of the studied genes reflected the typical polymorphism patterns of the local population.

### 2.4. Cytokine Analysis

Cytokine measurements during the exacerbation of HIV-related skin disorders were obtained for only 62 out of the 115 patients enrolled in this study. This limitation was primarily due to logistical and technical constraints, which consequently reduced the sample size for cytokine analysis and may have affected the broader applicability of the findings.

IL-1β and IFN-γ were selected as markers of immunological status due to their roles as pro-inflammatory cytokines, which are primarily produced by activated monocytes and macrophages and have wide-ranging immunological effects. IL-10—a key anti-inflammatory cytokine—was included for its critical role in modulating the immune response, notably as a potent inhibitor of Th1 cytokines such as IL-2 and IFN-γ.

Cytokine levels in plasma were measured using the MILLIPLEX^®^ MAP system (Austin, Texas, United States), which employs Luminex^®^ xMAP^®^ technology. This advanced multiplex platform is widely recognized in the life sciences for its ability to perform diverse bioassays, including immunoassays, using fluorescently encoded magnetic beads (MagPlex^®^-C microbeads, Austin, Texas, United States). Venous blood was collected in two sterile EDTA tubes. One tube was centrifuged immediately after collection for 15 min to separate the plasma, which was then analyzed for IL-1β, IL-10, and IFN-γ levels.

For the control group, data on 80 healthy individuals were obtained from the gene bank of RSU/JLCII.

### 2.5. Statistical Data Analysis

The Shapiro–Wilk test and normal Q–Q plots were used to evaluate data distribution, while Levene’s test was applied to assess variance homogeneity. Associations between quantitative variables (e.g., IL-10, IFN-γ, IL-1β, years to skin manifestation, CD4+ count, HIV-1 RNA load) and dichotomous variables (protective/risk alleles) were analyzed using the rank biserial correlation test. Spearman’s correlation test was utilized to examine relationships among cytokine levels, the time to manifestation of HIV-related disorders, CD4+ cell count, and HIV-1 RNA load. The chi-squared test of association was used to evaluate relationships between alleles. The Mann–Whitney U-test was used to compare plasma cytokine levels between the research and control groups, as well as the expression of cytokines between patients with the HLA DQA1*0101:0501 (risk) and 0501:0501 (protective) alleles.

To address the limited sample size of cytokine measurements, binomial logistic regression analysis with bootstrapping (1000 resamples) was conducted to evaluate the predictive role of cytokine levels (IL-1β, IFN-γ, IL-10) and HLA alleles regarding the likelihood of belonging to the group of PLWH with HIV-associated dermatoses compared to healthy controls. Stepwise forward and backward regression methods were applied, and the Akaike Information Criterion (AIC) was used to select the best-fitting model.

All statistical analyses were performed using the Jamovi software (v.2.3), and the results were considered statistically significant at *p* < 0.05.

## 3. Results

### 3.1. Comparison of Dermatological Groups and Immunological Parameters

All patients in the research group were selected based on the presence of HIV-related skin disorders, excluding opportunistic skin conditions and neoplasia. The skin pathologies were divided into two groups. Skin group I included patients with pruritic, papulosquamous, and seborrheic dermatoses (atopic dermatitis, prurigo, eosinophilic dermatitis, psoriasis, seborrheic dermatitis, acne, etc.), while skin group II included patients with secondary infections associated with skin dryness (microbial eczema, skin mycoses, localized and generalized pyoderma, and so on). In the research group, HIV-related skin disorders were first identified within 3 to 11 years following the diagnosis of HIV-1 infection, with an average onset time of 6 years.

The average number of dermatological conditions diagnosed over the entire observation period for PLWH was two.

No statistically significant differences were found between the two groups of dermatological conditions in terms of immunological parameters during the exacerbation of dermatological disorders (all *p*-values > 0.05), including viral load, CD4+ cell count, and plasma cytokine levels (Table 1).

### 3.2. Polymorphism of HLA Class II Alleles

To reduce variability in ethnic and geographic factors, participants in both the research and control groups were chosen from the same region, with no familial connections among them. The control group included healthy individuals whose families had lived in the region for at least three generations. Accordingly, the allele distribution of the studied genes represents the region’s typical genetic characteristics.

As mentioned in the previous publication of our study [25], we analyzed the polymorphism of HLA class II alleles by comparing allele frequencies between the research and control groups and identifying protective and risk alleles at the HLA-DRB1, HLA-DQA1, and HLA-DQB1 loci.

In the HLA-DRB1 locus, the alleles HLA-DRB1*07:13, DRB1*01:13, and DRB1*04:11 were observed less frequently in the research group than in the control group, suggesting their potential protective effect in the case of HIV infection with respect to HIV-associated skin disorders.

The frequency analysis of HLA-DQA1 alleles revealed distinct effects. The HLA-DQA1*0101:0501 allele was identified as a risk factor associated with a higher likelihood of the development of HIV infection with HIV-related dermatoses. Conversely, the HLA-DQA1*0501:0501 allele was more commonly found in the control group, indicating a potential protective effect in the case of HIV infection with HIV-associated skin disorders.

Protective and risk associations were also evident in the HLA-DQB1 locus. The HLA-DQB1*0201:0301 allele was detected more frequently in the research group, suggesting its association with increased susceptibility to HIV infection with HIV-related skin disorders (Table 2).

### 3.3. Association of CD4+ Cell Count and Other Markers in the Research Group

Our analysis revealed several significant associations between CD4+ cell counts and other markers in the research group.

The CD4+ cell count at the onset of HIV infection showed a significant inverse correlation with the viral load at the onset of HIV (r = −0.24, n = 115, *p* < 0.05) and a significant direct correlation with the interval to the development of HIV-related skin disorders (r = 0.3, n = 115, *p* < 0.01). Similarly, the HIV RNA load at the time of HIV onset demonstrated a significant negative correlation with the time to the manifestation of HIV-related skin pathologies (r = −0.25, n = 115, *p* < 0.01). Among the identified protective and risk alleles, only DRB1*07:13 (protective; r = 0.2, n = 115, *p* < 0.05) and DQB1*0201:0301 (risk; r = 0.2, n = 115, *p* < 0.05) were significantly associated with the viral load at the onset of HIV infection. The DQB1*0201:0301 risk allele was negatively associated with the CD4+ cell count at the time of HIV-associated skin pathology detection (r = −0.2, n = 115, *p* < 0.05).

In PLWH with HIV-related dermatoses, the IL-10 level was positively associated with the presence of the DQB1*0201:0301 risk allele (r = 0.26, n = 62, *p* < 0.05). A significant positive correlation was observed between IL-10 and IFN-γ levels (r = 0.52, n = 62, *p* < 0.001), as well as between IL-10 and the viral load at the time of onset of skin disorders (r = 0.3, n = 62, *p* < 0.05) in this group of patients.

### 3.4. Relationship Between Immunogenetic Biomarkers in the Research and Control Groups

Spearman correlations in both the research and control groups were determined to highlight the relationships between demographic factors, clinical parameters, and immunogenetic markers (Table 3). Among PLWH with a history of tuberculosis (TB), a significant negative correlation was observed with IL-1β and IL-10 levels, indicating lower interleukin levels in PLWH with skin disorders and a history of TB. Additionally, these patients showed a positive correlation with the DQA1*0101:0501 allele, suggesting potential genetic associations.

Age was positively correlated with IL-1β and IL-10 levels, suggesting that cytokine levels may increase with age. Conversely, age was negatively correlated with post-TB conditions in PLWH with skin disorders, indicating that younger patients may have had a higher prevalence of TB in the past.

DQA1*0101:0501 was negatively associated with IL-1b and IL-10, suggesting potential immunogenetic links. Other alleles showed weaker correlations with cytokines and clinical factors.

### 3.5. Expression Levels of Cytokines

**Figure 1:**
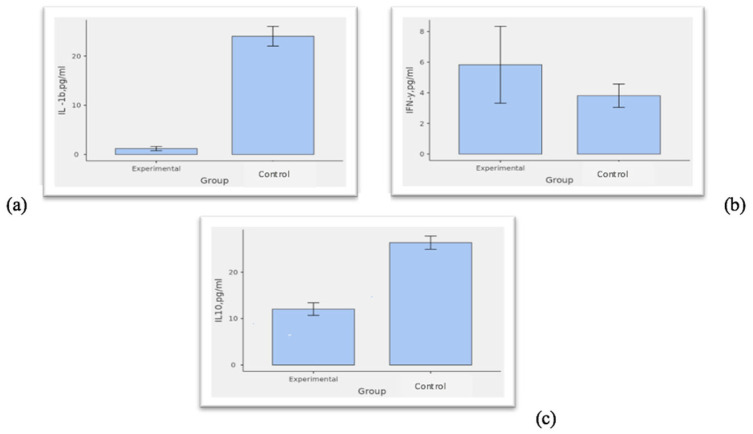
Differences between plasma cytokine levels in the research and control groups. Note: Comparison of (**a**) IL-1b levels; (**b**) IFN-y levels; and (**c**) IL-10 levels between the research and control groups. Abbreviations: pg/mL, picograms per milliliter.

Our results indicated statistically significant differences in plasma cytokine levels between the research and control groups (Figure 1).

### 3.6. Relationships Between Cytokines in the Research and Control Groups

In the research group of PLWH with dermatological conditions, a statistically significant positive correlation was identified between the anti-inflammatory cytokine IL-10 and the pro-inflammatory cytokine IFN-γ. In contrast, in the control group of healthy individuals, no correlation was observed between IL-10 and IFN-γ. However, a positive correlation was noted between IL-10 and IL-1β in the control group (Table 4).

### 3.7. Associations Between DRB1, DQA1, and DQB1 Alleles and Cytokine Responses

A comparison of cytokine expression between alleles revealed no statistically significant results for most loci. Specifically, the DRB1 and DQB1 alleles did not show significant correlations with cytokine expression levels.

However, alleles of the DQA1 gene demonstrated an association with IL-1β and IL-10 expression (Table 5).

The observed associations between the protective allele HLA-DQA1*0501:0501 and IL-1β and IL-10 suggest that this allele may confer an advantage in modulating the immune response. The high level of IL-1β in patients with the protective allele may indicate its role in enhancing the immune response, while an elevated level of IL-10 in these patients may be associated with its anti-inflammatory effect.

In contrast, the risk allele DQA1*0101:0501 is associated with lower levels of these cytokines, which may contribute to increased susceptibility to HIV infection with HIV-associated dermatoses.

### 3.8. Predictive Role of Cytokines in HIV-Related Dermatoses

To investigate whether cytokines can predict PLWH with HIV-related dermatoses, a binomial logistic regression analysis with bootstrapping (1000 resamples) was performed. The analysis controlled for the DQB1*0201:0301; DQA1*0501:0501 and 0101:0501; and DRB1*07:13, 01:13, and 04:11 alleles, as well as IL-1b, IFN-γ, IL-10, and gender. Only the best-fitting model is presented in the results (Table 6).

The result of the regression analysis aligns with the correlation analysis, confirming the role of IL-1β as a key factor, and revealing that IL-1β (Exp(B) = 0.76, *p* < 0.001) and IFN-γ (Exp(B) = 1.06, *p* = 0.043) are significant predictors for the likelihood of belonging to the group of PLWH with HIV-associated dermatoses. An increase in the IL-1β level by one unit decreased this likelihood by 24%, while an increase in the IFN-γ level by one unit increased it by 6%. No significant predictive role was observed for HLA alleles (*p* > 0.05), which may be attributed to the limited sample size and low allele frequency.

## 4. Discussion

The primary objective of our research was to investigate immunological and genetic factors and their correlation to HIV infection with HIV-associated skin diseases.

### 4.1. Predictive Role of Cytokines

Our findings highlight the critical roles of cytokines in modulating disease progression. In PLWH with HIV-related dermatological conditions, a statistically significant positive correlation was observed between IL-10 and IFN-γ levels; furthermore, elevated IFN-γ levels may be associated with a higher risk of developing pathological conditions. Previous studies have similarly reported elevated IL-10 levels in PLWH [26], with IFN-γ levels also being significantly higher in PLWH with psoriasis compared to healthy controls [27]. IL-10 levels have been shown to correlate with higher HIV viral loads [28]. A positive correlation between IL-10 and IFN-γ levels in patients with HIV-related skin disorders has been noted in studies involving infants with HIV [29].

The IFN-γ/IL-10 ratio has been investigated in various dermatological studies as a potential predictive biomarker. Research on metastatic melanoma patients undergoing anti-PD-1 therapy identified a higher IFN-γ/IL-10 ratio in responders, suggesting its potential as a predictive tool for therapeutic outcomes [30].

Studies have reported elevated IFN-γ levels in PLWH with psoriasis compared to HIV-negative psoriasis controls, indicating a distinct immunological profile in HIV-related psoriasis [31,32]. The connection between IFN-γ and dermatoses can be attributed to its role in enhancing cell-mediated cytotoxicity against keratinocytes, which is achieved by inducing MHC class I expression in keratinocytes through a JAK2/STAT1-dependent pathway [33].

Our research confirmed the critical roles of IL-1β and IFN-γ in the pathogenesis of HIV infection with HIV-associated dermatoses. We may assume that elevated IL-1β levels appear to reduce the likelihood of developing these conditions, potentially reflecting IL-1β’s role in modulating inflammatory responses. Conversely, increased IFN-γ levels are associated with a higher likelihood of HIV infection with HIV-related dermatoses, consistent with its pro-inflammatory and immune-activating properties. These results are in agreement with earlier research highlighting the role of IL-1β in the development of neutrophilic dermatoses, as well as the contribution of IFN-γ to keratinocyte apoptosis and disturbances in immune regulation [34,35].

### 4.2. HLA

Our earlier study revealed a higher frequency of the DQB1*0201:0301 allele in the HIV-related group, with this risk allele being significantly associated with higher HIV RNA loads. Conversely, the DQA1*0101:0501 allele was identified as a risk factor for increased susceptibility to HIV infection with HIV-related dermatoses [25]. Additionally, the DQB1*03:01 and 05:01 alleles were more common in PLWH than in healthy controls [36].

We found an association between the protective HLA-DQA1*0501:0501 allele and IL-1β and IL-10 expression. This suggests that IL-10 may act as an anti-inflammatory cytokine in conjunction with the HLA-DQA1*0501:0501 allele, potentially modulating the pro-inflammatory effects of IL-1β. It is well-established that IL-1α and IL-33 function as “dual-function” cytokines, and IL-37 possesses strong anti-inflammatory and immunoregulatory properties [37,38].

Based on Spearman’s correlation analysis, we observed an association between the HLA-DRB1*07:13 allele and lower viral loads, as well as between the HLA-DQB1*0201:0301 allele and higher viral loads and lower CD4+ T cell counts.

These findings are consistent with previous studies indicating that HLA-DQB1*02:01 is associated with severe immunosuppression, contributing to poorer clinical outcomes. Additionally, HLA-DQB1*03:02 has been linked to accelerated disease progression in PLWH, particularly in specific populations such as African Americans [39].

A study investigating HLA alleles as predictors of response to interferon therapy in HCV patients demonstrated that the HLA-DRB1*07-13 allele was more frequently found in healthy controls, suggesting that this allele may enhance the immune response against HCV [40].

### 4.3. Clinical Implications

The lack of significant predictive value for HLA alleles in our study highlights the need for larger sample sizes in order to better understand their roles in cytokine regulation and disease susceptibility.

The predictive roles of IL-1β and IFN-γ in HIV-related dermatoses suggest potential targets for therapeutic interventions. IL-1β may serve as a modulator of inflammatory responses, reducing disease susceptibility, while IFN-γ’s pro-inflammatory properties highlight its role in disease progression. The association between HLA alleles and cytokine profiles underscores the need for personalized approaches to managing HIV-related skin disorders.

### 4.4. Limitations

Our study findings are subject to certain limitations. First, limited cytokine measurements may affect the generalizability of the results. While the method of binomial logistic regression enhances the reliability of statistical associations through the estimation of variability and confidence intervals, it cannot fully compensate for the reduced availability of cytokine data, which may limit the robustness of certain conclusions. Furthermore, the cytokine data were collected at a single time point without longitudinal follow-up. As such, dynamic changes in cytokine levels that occur during disease progression could not be accounted for. The low frequencies of certain HLA alleles reduced their predictive significance. Therefore, further research is needed to validate these findings and explore their implications for clinical practice.

## 5. Conclusions

Our findings suggested that specific HLA class II alleles may play a role in modulating the relationship between HIV infection and the development of HIV-associated skin disorders. Based on the observed associations, we can hypothesize that individuals with the protective DRB1*07:13 allele may demonstrate enhanced immune resilience, as reflected in reduced viral loads, elevated CD4+ counts, and the later onset of skin conditions. In contrast, individuals carrying the risk allele DQB1*0201:0301 are more likely to exhibit an earlier onset of skin conditions, elevated viral loads, and decreased CD4+ cell counts.

Our data suggest that while IL-10 is classically considered an anti-inflammatory Th2 cytokine, patients carrying the HLA-DQB1*0201:0301 risk allele in our research group showed an association between IL-10 levels and pro-inflammatory activity. This observed correlation may also be linked to the timing of skin disease manifestations following the onset of HIV. Further studies are needed to confirm causality and underlying mechanisms.

We found that the protective DQA1*0501:0501 allele is associated with IL-1β and IL-10 responses, which may contribute to reduced susceptibility to the progression of HIV infection with HIV-associated dermatoses. These cytokines, in conjunction with this allele, are likely involved in modulating inflammatory processes. Conversely, the risk allele HLA-DQA1*0105:0501 was linked to lower IL-1β levels, potentially increasing susceptibility to these conditions. The roles of IL-1β and IFN-y as predictors of HIV infection with HIV-related dermatoses highlight their importance in the progression of the disease. These findings emphasize the interplay between HLA class II genes and cytokine levels in determining genetic risk and clinical outcomes in PLWH with HIV-related dermatoses. Further research is required to elucidate the mechanisms through which cytokines and HLA polymorphisms influence the progression of HIV-associated dermatoses, particularly in the Latvian population.

## Figures and Tables

**Table 1:** Comparison of viral load and immunological parameters between two groups of dermatological disorders in PLWH.

Parameter	Skin Group I (n = 77) Median (IQR)	Skin Group II (n = 38) Median (IQR)	*p*-Value
HIV RNS III, copy/mL	81.00 (20.00–131,000.00)	55.00 (20.00–126,250.00)	0.949
CD4+ III, cells/mL	222.00 (80.00–364.00)	196.00 (86.25–373.00)	0.704
IL-1b, pg/mL	0.00 (0.00–0.00)	0.00 (0.00–0.00)	0.543
IFN-y, pg/mL	0.00 (0.00–3.50)	0.00 (0.00–1.00)	0.831
IL-10, pg/mL	8.00 (6.00–12.90)	9.10 (4.00–14.90)	0.813

Note. H_a_ μ skin group I ≠ skin group II; Abbreviations: CD4+ III, CD4+ cell counts at the time of skin disease exacerbation; HIV RNS III, HIV viral load at the time of skin disease exacerbation; pg/mL, picograms per milliliter.

**Table 2:** The distribution of protective and risk alleles of HLA class II in the research (n = 115) and control (n = 80) groups [25], reproduced with permission from A. Soha et al., Acta Dermatovenerol APA, published by the Association of Slovenian Dermatovenerologists, 2024.

Alleles	Experimental Relative Frequency	Control Relative Frequency	OR (95% CI)	*p*
DRB1 07-13	0.02	0.09	0.19 (0.04–0.91)	0.022
DRB1 01-13	0.01	0.09	0.09 (0.01–0.76)	0.006
DRB1 04-11	0	0.05	0.07 (0.001–1.39)	0.015
DQA1 0101-0501	0.22	0.06	4.2 (1.5–11.4)	0.003
DQA1 0501-0501	0.03	0.10	0.24 (0.06–0.94)	0.028
DQB1 0201-0301	0.10	0	19.4 (1.1–333)	0.003

Note: OR = odds ratio; CI = confidence interval.

**Table 3:** Correlation analysis of demographic factors and immunogenetic markers in both the research and control groups.

		1	2	3	4	5	6	7	8	9	10	11	12
1	Age	—											
2	Gender	0.105	—										
3	Post TB	−0.307 ***	−0.013	—									
4	IL-1b	0.295 ***	−0.096	−0.697 ***	—								
5	IFN-y	0.089	0.116	−0.182 *	0.169 *	—							
6	IL10	0.219 **	−0.077	−0.566 ***	0.591 ***	0.225 **	—						
7	DQA1*0101:0501	−0.106	0.064	0.258 ***	−0.260 **	−0.077	−0.247 **	—					
8	DQA1*0501:0501	0.076	0.039	−0.137	0.192 *	−0.009	0.153	−0.102	—				
9	DRB1*07:13	0.003	0.007	−0.114	0.103	0.015	0.120	−0.092	0.158 *	—			
10	DRB1*01:13	0.037	0.043	−0.101	0.084	−0.110	0.110	−0.014	−0.051	−0.045	—		
11	DRB1*04:11	−0.051	0.030	−0.108	0.054	0.164	0.033	−0.060	−0.035	−0.032	−0.030	—	
12	DQB1*0201:0301	−0.130	0.008	0.209**	−0.237 **	−0.019	−0.056	−0.107	−0.063	−0.056	−0.053	−0.037	—

Note. * *p* < 0.05, ** *p* < 0.01, *** *p* < 0.001.

**Table 4:** Relationships between cytokines in the research and control groups.

		Research (n = 115)			Control (n = 80)	
	IL-1b, pg/mL	IFN-y, pg/mL	IL10, pg/mL	IL-1b, pg/mL	IFN-y, pg/mL	IL10, pg/mL
IL-1b, pg/mL	-			-		
IFN-y, pg/mL	0.059	-		0.101	-	
IL10, pg/mL	0.189	0.518 ***	-	0.402 ***	−0.097	-

Note. *** *p* < 0.001.

**Table 5:** Comparison of expression of cytokines in participants with HLA DQA1 0101-0501 (risk) and 0501-0501 (protective) alleles.

Parameter	DQA1 0101-0501	DQA1 0501-0501	*p*-Value	Effect Size
	Median (Q1–Q3)	Median (Q1–Q3)		
IL-1b, pg/mL	0.00 (0.00–0.00)	28.75 (19.10–32.60)	< 0.001	0.79
IFN-y, pg/mL	0.00 (0.00–3.50)	0.00 (0.00–3.85)	0.677	NA
IL-10, pg/mL	9.10 (4.80–16.00)	31.30 (14.43–41.75)	0.010	0.63

Note: NA, not applicable. Abbreviations: pg/mL, picograms per milliliter.

**Table 6:** Binomial logistic regression models predicting the role of cytokines in PLWH with HIV-associated dermatoses.

Exp(B) 95% Confidence Intervals							
Name	Estimate	SE	Exp(B)	Lower	Upper	z	*p*
(Intercept)	−2.4040	0.6611	0.0904	0.00484	0.237	−3.64	<0.001
IL-1b, pg/mL	−0.2683	0.0542	0.7647	0.62555	0.824	−4.95	<0.001
IL-10	−0.0702	0.0294	0.9322	0.81632	0.991	−2.38	0.017
IFN-y	0.0634	0.0313	1.0654	0.98488	1.176	2.02	0.043

Note: Exp(B), Odds Ratio.

## Data Availability

Public repository name: Rīga Stradiņš University Institutional Repository Dataverse. Dataset accession number(s) or DOI(s): Soha, Alena; Azina, Inga; Zolovs, Maksims; Miskina, Darja Arina; Murasko, Viktorija; Rozentale, Baiba; Hartmane, Ilona; Rubins, Andris, 2025, “HLA Class II, Cytokines, HIV”, https://doi.org/10.48510/FK2/VD71HQ, Rīga Stradiņš University Institutional Repository Dataverse [41], V1, UNF:6:90ylph76WaJa1euf+j9oBQ==[fileUNF]; Link(s) to the script/code repository: https://dataverse.rsu.lv/dataset.xhtml?persistentId=doi:10.48510/FK2/VD71HQ (accessed on 5 February 2025).

## Abbreviations

The following abbreviations are used in this manuscript:

AMPs	antimicrobial peptides
ART	antiretroviral therapy
AD	atopic dermatitis
EBV	Epstein-Barr virus
IFN-γ-iMSC-EVs	IFN-y-induced mesenchymal stem cell extracellular vesicles
DCs	dendritic cells
HLA	human leucocyte antigen
RSY/JLCII	the Joint Laboratory of Clinical Immunology and Immunogenetics at Riga Stradiņš University
PPE	pruritic papular eruption
PLWH	patients living with HIV/AIDS
SD	seborrheic dermatitis
TB	tuberculosis
